# Prospective assessment of key factors influencing treatment strategy and outcome of fragility fractures of the pelvis (FFP)

**DOI:** 10.1007/s00068-022-01887-1

**Published:** 2022-02-05

**Authors:** Pol Maria Rommens, Johannes Christoph Hopf, Charlotte Arand, Kristin Handrich, Mehdi Boudissa, Daniel Wagner

**Affiliations:** Department of Orthopaedics and Traumatology, University Medical Center, Langenbeckstrasse 1, 55131 Mainz, Germany

**Keywords:** Pelvis, Fragility fracture, Prospective, Conservative, Operative, Outcome, Delayed hospitalization, Mortality, Complications

## Abstract

**Background:**

Fragility fractures of the pelvis (FFP) are a clinical entity with an increasing significance in clinical practice. Little is known about the conditions, which influence decision making and outcome.

**Setting:**

Level I trauma center.

**Material and methods:**

Prospective assessment of selected parameters of patients, who were admitted with a FFP in a 2-year period. Fractures were classified in accordance with the Rommens and Hofmann classification. Living environment, level of autonomy (independent walking), type of treatment (conservative versus operative), type of surgical technique, European Quality of Life-5 Dimensions-5 Levels (EQ-5D-5L), Short Form-8 Physical Component Score (SF-8 PCS) and Short Form-8 Mental Component Score (SF-8 MCS), Barthel Index, Parker Mobility Score (PMS) and Numeric Rating Scale (NRS) were collected at primary presentation (t1), at discharge (t2) and after 3 (t3) and 12 months (t4). Length of hospital stay, in-hospital complications, surgery-related complications, new osteoporotic fractures and mortality rate within the first year were also registered. The key factors influencing the choice of therapy and outcome were looked for.

**Results:**

110 patients, 99 women (90%) and 11 men (10%), were included in the study. Their mean age was 79.2 years (SD 10 years). Fourteen patients had FFP type I (12.7%), 59 FFP type II (53.6%), 11 FFP type III (10%) and 26 FFP type IV fractures (23.6%). All patients with FFP type I were treated conservatively. 48 patients with FFP types II-IV were treated conservatively and 48 operatively. Patients, who got a conservative outpatient treatment first and were hospitalized later, had higher FFP fracture types at admission. Operatively treated patients were hospitalized at a median of 33.5 days after the beginning of complaints, whereas the median day of admission of the conservative group was the day of trauma (*p* < 0.001). The operatively treated patients were hospitalized in a worse clinical condition (SF-8 PCS, EQ-5D-5L, autonomy). Length of stay (LoS) of operatively treated patients was significantly longer than of conservatively treated (*p* < 0.001). There was a tendency to more in-hospital complications in the operative group (*p* = 0.059). The rate of surgery-related complications (8.3%) was low with only one revision needed. Selected outcome parameters improved during the observation period nearly reaching the level before FFP after 1 year. SF-8 PCS, Barthel index and rate of patients living home were higher in the operative group at t4. The improvement of autonomy (independent walking) between t1 and t4 was significant in the operated group (*p* = 0.04) but not in the conservative group (*p* = 0.96). One-year mortality rate was 11.7% with no difference between the fracture types. One-year mortality rate of conservatively treated patients with FFP type II-IV was 13.5% versus 6.9% in the operative group (*p* = 0.38).

**Conclusion:**

Conservative treatment is appropriate in patients with FFP type I as well as in patients with FFP type II, provided that the last ones are hospitalized immediately after the traumatic event. Surgical treatment is recommended in patients with higher fracture types, with delayed presentation or after unsuccessful conservative treatment. In the conservative and operative group, all selected parameters considerably improved between t1 and t4 with a steeper increase in the operative group. The rate of postoperative complications is low. The 1-year mortality rate is the lowest in the operative group. Surgical stabilization of FFP is safe and reliable provided it is performed with care and in the appropriate target group.

## Introduction

There is a growing clinical-scientific interest in fragility fractures of the pelvis (FFP) [1]. The incidence of this emerging pathology is increasing due to higher life expectancy and high rates of osteoporosis in elderly women [2, 3]. The characteristics of FFP are not comparable to those of pelvic fractures in younger patients. Not only are trauma mechanisms completely different, also clinical symptoms, fracture patterns and natural course are unique and diverse [4, 5]. Clinical data on the origin, diagnosis, treatment strategy and outcome of FFP become increasingly available, but there still is uncertainty and controversy on how to manage these lesions [6–8]. Several authors bring arguments for conservative, others for operative treatment [9–11]. Osterhoff et al. state that patients with FFP are of old age and present with several comorbidities. Conservative treatment is the least invasive. Adequate pain therapy enables quick mobilisation and early discharge from hospital. Operatively treated patients stay longer in the hospital. A long hospital-stay enhances the risk of general complications such as urinary tract infection, pneumonia or bedsores. Surgical treatment is more invasive and may be connected with complications such as hematoma, infection, malposition or loosening of implants [9, 10]. On the contrary, Wagner et al. described lower mortality and better mobility after surgical stabilization [11]. Nevertheless, Rommens et al. found out that open surgical procedures are responsible for more complications than less-invasive stabilisation techniques [10].

The FFP-classification provides a frame for analysis of these lesions. It is based on the analysis of conventional radiographs and pelvic CT-data of 245 patients with FFP. The classification distinguishes between four different levels of instability. Patients with FFP type I have isolated anterior pelvic ring fractures. Patients with FFP type II have non-displaced posterior pelvic ring lesions. Patients with FFP type III have unilaterally displaced posterior ring lesions and patients with FFP type IV have bilaterally displaced posterior ring lesions [12]. The intra-rater and inter-rater reliabilities of the FFP-classification have been validated in a multicentre study [13]. In the original publication, the FFP-classification was connected with recommendations for surgical treatment. So far, they were not validated by prospective studies. Published studies merely focus on indications, timing and techniques of surgical stabilization [8, 9, 14, 15]. This prospective study investigates which factors influence decision making and outcome of patients with FFP.

## Materials and methods

**Fig. 1:**
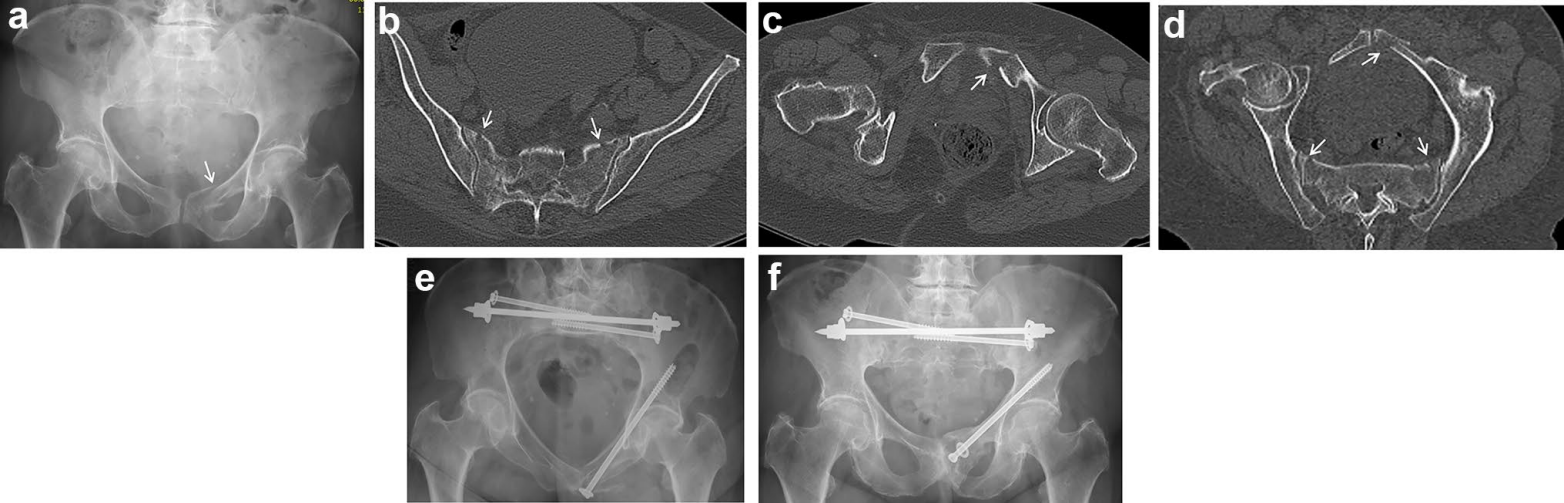
**a–f** A 77-year-old woman suffered severe pelvic pain after a fall at home. The a.p. pelvic overview shows a left superior and inferior pubic ramus fracture (arrow) (**a**). A transverse CT-cut through the sacrum shows bilateral non-displaced sacral alar fractures, complete on the right and incomplete on the left side (arrows) (**b**). Transverse CT-cut through the pubic symphysis confirms the left-sided superior pubic ramus fracture (arrow) (**c**). Oblique CT-cut though the level of the pelvic brim shows the posterior and anterior instabilities of the pelvic ring (arrows) (**d**). These fractures corresponded with a FFP type IIc. The patient was treated conservatively during 1 week. Due to continuing immobilizing pain, surgical stabilization was performed after 8 days. The fractures were fixed operatively with a transsacral bar and bilateral iliosacral screws. The pubic ramus fracture was stabilized with a retrograde transpubic screw. Postoperative pelvic inlet view (**e**). The a.p. pelvic overview more than one year after surgery shows complete healing of all fractures. There is a slight loosening of the retrograde transpubic screw (**f**)

We prospectively collected demographics and medical history of all patients, who were admitted at our department between mid-2018 and mid-2020 (2-year period) with the diagnosis of fragility fractures of the pelvis (FFP). FFP were diagnosed by means of anamnesis, conventional pelvic radiographs (a.-p., inlet and outlet) and pelvic computer tomography (CT) and classified by the first and last author in accordance to the FFP-classification [12]. The quality of life (QoL) before the fracture was retrospectively collected with the Short Form-8 Physical and Mental Component Scores (SF-8 PCS and SF-8 MCS) [16] and with the European Quality of Life-5 Dimensions-5 Levels (EQ-5D-5L) questionnaire [17, 18] at hospital admission. The following data are additionally collected at admission (timepoint 1 = t1): age, sex, trauma mechanism, start of complaints in case no trauma was memorable, comorbidities, EQ-5D-5L, level of autonomy (independent walking, walking with sticks, walking with walking frame or rollator, bedridden), most recent living environment, Barthel index [19], Parker Mobility Score (PMS) [20] and Numeric Rating Scale on load (NRS) [21]. A comorbidity was defined as a known disease with which the patient is admitted. In accordance with the recommendations of Rommens and Hofmann [12, 22], conservative treatment was given for patients with FFP type I and FFP type II. Surgical stabilization was recommended for patients with FFP type III and FFP type IV. Surgical stabilization was also recommended after 5–7 days for patients with FFP type II in case of unsuccessful conservative treatment (Fig. 1a–f).

At discharge (timepoint 2 = t2), the following data are collected: type of management (conservative versus operative), type of surgical stabilization, length of hospital stay (LoS), general in-hospital complications, surgery-related complications, in-hospital mortality, level of autonomy and destination. Additionally, the following scores were collected: SF-8 PCS, SF-8 MCS, EQ-5D-5L, Barthel index, PMS and NRS.

Patients or their relatives were contacted by phone 3 months (timepoint 3 = t3) and 12 months (timepoint 4 = t4) after primary admission. Their general practitioner or the bureau of vital statistics was contacted to ask about their vital status, if patients were not directly available. The following data were collected at t3 and t4: actual living environment, level of autonomy, mortality, SF-8 PCS and SF-8 MCS, EQ-5D-5L, Barthel index, PMS and NRS. The number of new fragility fractures and new hospitalizations within the first year were also noticed.

All included patients or their relatives gave their written approval for participation in the study, data collection and analysis. Personal data were anonymized before analysis. The study was approved by the local ethics committee (Reference: 837.140.17 (10974)).

We tested continuous data for normal distribution using the Kolmogorov Smirnov test. Descriptive statistics in normally distributed data were described as mean and standard deviation. In non-normally distributed data, median and the 25th and 75th interquartile ranges (IQR) were calculated. Different groups were compared using the non-paired student’s *t* test (normally distributed data) and the Mann–Whitney-*U* test (non-normally distributed data). Nominal groups were compared using the chi-square test. A *p* value of ≤ 0.05 was considered to be significant. Statistical analysis was performed using SPSS software (IBM SPSS Statistics for Windows, Version 23; IBM Corp, Armonk, NY, USA).

## Results

### All patients

**Fig. 2:**
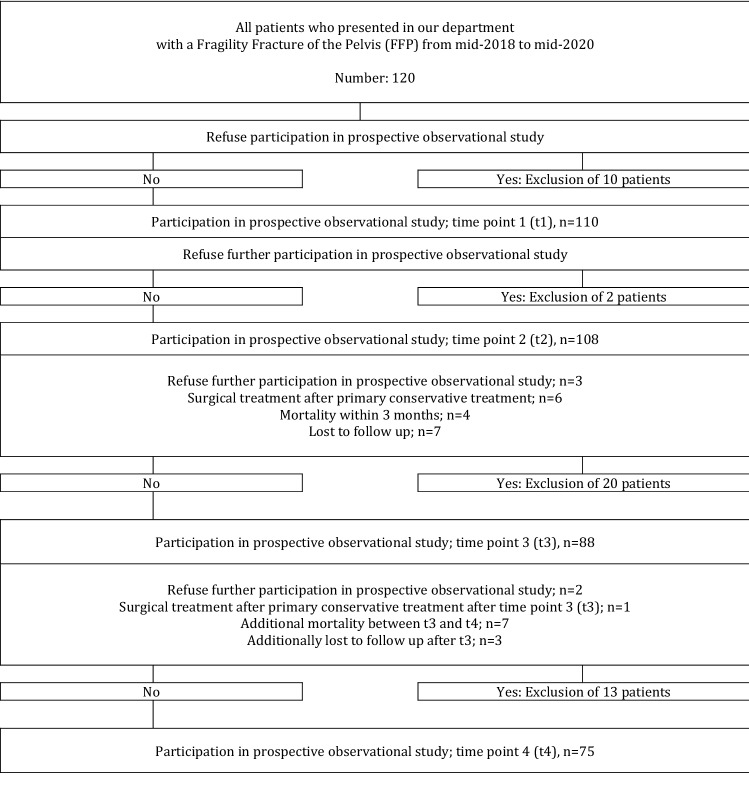
Flowchart of included and excluded patients during the course of the study

110 were included in the study (t1). At t2, there were 108 patients left with complete documentation, at t3 88 and at t4 75 patients. The reasons for exclusion during the course of the study are depicted in the Fig. 2. The mean age of all 110 patients was 79.2 years (SD 10 years). There were 99 women (90%) and 11 men (10%). Before hospital admission, 94 patients (85.5%) lived independently or with assistance in their own home. Fourteen patients had FFP type I (12.7%), 59 FFP type II (53.6%), 11 FFP type III (10%) and 26 FFP type IV fractures (23.6%). 99 patients (90%) presented with comorbidities. 63 patients had a history of osteoporosis (57.3%), 40 already suffered another fragility fracture before suffering FFP (36.4%) and only 38 received and antiresorptive medication (34.5%). Only 20% of patients could walk independently at t1. 62 patients were treated conservatively (56.4%) and 48 operatively (43.6%). The frequency and type of surgical techniques for stabilization of the posterior and anterior pelvis, depending on the FFP-classification, are shown in Table 1. With the exception of plate and screw osteosynthesis for fractures of the posterior pelvis (*n* = 3) and the anterior pelvis (*n* = 9), all procedures were performed minimally invasive. Median LoS was 11 days (min 3-max 42 days, IQR 8–17 days). There were general in-hospital complications in 29 patients (26.9%). There were surgery-related complications in 4 patients (4/48 = 8.3%) and only one surgical revision was needed (1/48 = 2.1%). There was no in-hospital mortality. The one-year mortality rate was 11.7% for the whole group. Between t2 and t4, 11 patients suffered an additional osteoporotic fracture (14.7%) and 27 patients were re-hospitalized for any reason (36.0%). Demographics and selected parameters of all patients during hospitalization and during the course of the observation period are depicted in Table 2. The evolution of the patient-reported outcomes is depicted in Table 3.

**Table 1:** Frequency and type of surgical techniques for stabilization of the posterior and anterior pelvis, depending on FFP-classification, surgery-related complications and revisions, secondary operative treatment

FFP-categories	FFP types I–IV	FFP type I	FFP type II	FFP type III	FFP type IV
Number of operatively treated patients	48	0	14	10	24
**Posterior Pelvis**					
Transsacral bar with bilateral iliosacral screws	16	0	4	0	12
Transiliac internal fixator with bilateral iliosacral screws	10	0	2	0	8
Transiliac internal fixator with unilateral iliosacral screw	7	0	3	3	1
Transsacral bar with unilateral iliosacral screw	3	0	1	1	1
Plate and screw osteosynthesis ilium	3	0	0	3	0
Transiliac internal fixator with bilateral transsacral screws	3	0	1	1	1
Iliosacral screw unilateral	2	0	1	1	0
Iliosacral screws bilateral	1	0	1	0	0
Screw osteosynthesis ilium unilateral	1	0	0	1	0
Transsacral bar	1	0	1	0	0
Bilateral transsacral screws with bilateral iliosacral screws	1	0	0	0	1
**Anterior Pelvis**					
Unilateral retrograde transpubic screw	17	0	3	6	8
Plate and screw osteosynthesis	9	0	1	1	7
Bilateral retrograde transpubic screw	4	0	3	1	0
Retrograde transpubic screw and plate and screw osteosynthesis	1	0	0	1	0
**Surgery-related complications** - **total**	4	*n.a.*	2	0	2
Malposition	2		2	0	0
Malposition and paresis	1		0	0	1
Plate breakage	1		0	0	1
**Surgical revisions**	1	*n.a.*	0	0	1
**Secondary operative treatment** (after primary conservative treatment) *	7/62	2/14	4/45	1/1	0/2

*The figures show the number of secondarily operated patients in relation to the total number of primarily conservatively treated patients

*n.a.* not applicable

**Table 2:** Demographics, type of treatment and selected data of all patients, of patients with FFP I and of conservatively and operatively treated patients with FFP type II–V during the course of the observation period

	All patients	FFP type I	FFP type II-IV conservative	FFP type II-IV operative	*p* value
Patients; *n* (%)	110 (100.0)	14 (12.7)	48 (43.6)	48 (43.6)	
Mean age (years)	79.2	81.3	80.9	76.8	**0.04**
Standard Deviatio*n* (SD) (years)	10.0	9.6	9.7	9.9	
Women; *n* (%)	99 (90.0)	11 (78.6)	41 (85.4)	47 (97.9)	0.059
Men; *n* (%)	11 (10.0)	3 (21.4)	7 (14.6)	1 (2.1)	0.059
Patients with comorbidities; *n* (%)	99 (90.0)	14 (100.0)	43 (89.6)	42 (87.5)	0.75
Patients with two or more comorbidities; *n* (%)	63 (57.3)	10 (71.4)	28 (58.3)	25 (52.1)	0.54
Osteoporosis in medical history; *n* (%)	63 (57.3)	7 (50.0)	25 (52.1)	31 (64.6)	0.21
Previous osteoporotic fracture; *n* (%)	40 (36.4)	5 (35.7)	15 (31.3)	20 (41.7)	0.29
Patients with more than 5 different drugs per day; *n* (%)	75 (68.2)	9 (64.3)	34 (70.8)	32 (66.7)	0.66
Anti-resorptive medication; *n* (%)	38 (34.5)	5 (35.7)	15 (31.3)	18 (37.5)	0.52
**Living situation before admission**; *n* (%)					
Independently at home	83 (75.5)	9 (64.3)	36 (75,0)	38 (79.2)	0.63
Assisted living at home	11 (10.0)	3 (21.4)	4 (8.3)	4 (8.3)	1
Nursing home	9 (8.2)	0 (0.0)	6 (12.5)	3 (6.3)	0.49
Hospital	7 (6.4)	2 (14.3)	2 (4.2)	3 (6.3)	1
**Trauma mechanism**; *n* (%)					
Fall from standing position	74 (67.3)	13 (92.9)	36 (75.0)	25 (52.2)	0.01
Recurrent falls	8 (7.3)	1 (7.1)	3 (6.3)	4 (8.3)	1
No trauma memorable	28 (25.5)	0 (0.0)	9 (18.8)	19 (39.6)	**0.02**
**Time between trauma or beginning of complaints and t1** (days)					
Median	4	0	0	33.5	** < 0.001**
Min	0	0	0	0	
Max	361	61	123	361	
**Type of primary treatment**;* n* (%)					
Conservative	62 (56.4)	14 (100.0)	48 (100.0)	0 (0.0)	1
Operative	48 (43.6)	0 (0.0)	0 (0.0)	48 (100.0)	1
**Median length of hospital stay** (days)	11	9	9	15	** < 0.001**
**Median length of postoperative hospital stay** (days)	*n.a*.	*n.a*.	*n.a*.	9	
**Patients with general in-hospital complications**, *n* (%)	29 (26.9)	5 (38.5)	8 (16.7)	16 (34.0)	0.059
Urinary tract infection; *n*	19	2	3	13	0.011
Pneumonia; *n*	9	1	4	4	1
Bedsore; *n*	3	0	1	3	0.62
**Patients with surgery-related complications**; *n* (%)	*n.a*.	*n.a*.	*n.a*.	3 (6.3)	
Implant malposition; *n*				3	
Paresis; *n*				1	
**Surgical revisions**; *n* (%)	*n.a*.	*n.a*.	*n.a*.	1 (2.1)	
Secondary operative treatment; *n* (%)	7 (6.4)	2 (14.3)	5 (10.4)	n.a	
One-year mortality rate; %	11.7	11.1	13.5	6.9	0.38
New osteoporotic fracture between t2 and t4; *n* (%)	11 (14.7)	2/7 (28.6)	2 (4.8)	7 (15.9)	0.157
Re-hospitalization rate between t2 and t4; *n* (%)	27 (36.0)	2/7 (28.6)	17 (48.7)	15 (33.6)	0.393

*n.a.* not available; *p* values < 0.05 are depicted in bold

**Table 3:** Patient-reported outcomes of all patients (*n* = 110) during the course of the observation period

	Before trauma	t1	t2	t3	t4
Patients living at home independently or with assistance (%)	85.5	*n.a*.	39.8	79.5	86.7
Short Form-8 Physical Component Score (median)	40.67	*n.a*.	23.92	32.78	36.77
Short Form-8 Mental Component Score (median)	57.25	*n.a*.	55.32	56.93	55.65
European Quality of Life-5 Dimensions-5 Levels (median)	0.86	0.16	0.34	0.76	0.72
Patients walking independently (%)	*n.a*.	20	4.6	23.8	28.0
Barthel index (median)	*n.a*.	45	65	85	90
Parker mobility score (median)	*n.a*.	1	2	4	5
Numeric rating scale on load (median)	*n.a*.	10	7	4	4

*n.a.* not available

### FFP type I

There were only 14 patients with FFP type I (isolated anterior pelvic fracture). Their mean age was 81.3 years (SD 9.6 years). There were 11 women (78.6%) and 3 men (21.4%). Twelve patients (85.7%) lived independently or with assistance in their own home. All patients presented with comorbidities. All patients were treated conservatively. Median hospital stay was 9 days (min 4-max 41 days, IQR 7–15 days). There were general in-hospital complications in 5 patients (38.5%). Between t2 and t4, 2 patients suffered a fracture progression and needed a surgical stabilization. Two additional patients suffered another osteoporotic fracture and needed re-hospitalization. At t4, four of the remaining patients lived at home (57.1%) but only one could walk without aid (14.3%). One-year mortality rate was 11.1%. Demographics and selected parameters of patients with FFP type I during hospitalization and during the course of the observation period are depicted in Table 2. The evolution of the patient-reported outcomes is depicted in Table 4.

**Table 4:** Patient-reported outcomes of patients with FFP type I (*n* = 14) during the course of the observation period

	Before trauma	t1	t2	t3	t4
Patients living at home independently or with assistance (%)	85.7	*n.a*.	30.8	37.5	57.1
Short Form-8 Physical Component Score (median)	47.62	*n.a*.	23.11	35.02	31.5
Short Form-8 Mental Component Score (median)	56.82	*n.a*.	61.94	51.09	55.53
European Quality of Life-5 Dimensions-5 Levels (median)	0.92	0.14	0.24	0.56	0.8
Patients walking independently (%)	*n.a*.	21.4	0.0	12.5	14.3
Barthel index (median)	*n.a*.	30	55	75	85
Parker Mobility Score (median)	*n.a*.	0	1	5	5
Numeric Rating Scale on load (median)	*n.a*.	10	7	4	4

*n.a.* not available

### FFP types II-IV

96 patients had FFP with involvement of the posterior pelvic ring (FFP type II, FFP type III or FFP type IV) (87.3%). Their mean age was 78.9 years (SD 10.0 years). There were 88 women (91.7%) and 8 men (8.3%). 74 patients (77.1%) lived independently or with assistance in their own home. 85 patients presented with comorbidities (88.5%). 48 patients were treated conservatively (50.0%) and 48 operatively (50.0%). Demographics and selected parameters of patients with FFP type II-IV during hospitalization and during the course of the observation period are depicted in Table 2. The evolution of the patient-reported outcomes is depicted in Table 5. There were several important differences between the conservative and operative group. Conservatively treated patients were hospitalized at the day of trauma, whereas operatively treated patients were admitted at a median of 33.5 days after beginning of complaints (*p* < 0.001). The clinical condition of the patients, who were treated operatively later on, was worse at admission: only 12.5% of the operative group could walk independently but 27.1% of the conservative group. Moreover, SF-8 PCS and EQ-5D-5L were lower in the operative group before trauma and at the time of hospitalization. Surgery was performed at a median of 6 days after admission and operatively treated patients stayed 6 days longer in hospital (15 versus 9 days) (*p* < 0.001). There was a tendency to more general in-hospital complications in the operative group (34.0% versus 16.7%) (*p* = 0.059), mainly because of urinary tract infection (*p* = 0.011). Surgery-related complications were seen in 4 patients (8.3%). Five patients of the conservative group (10.4%) needed a secondary operative stabilization because of fracture progression or unsuccessful conservative treatment. One-year mortality rate was 11.8% for all patients with FFP types II-IV, 13.5% for the conservative and 6.9% for the operative group (*p* = 0.38). After 1 year (t4), 92.3% of the operative and 86.2% of the conservative group lived at home. 30.8% of the operative versus 27.6% of the conservative group regained full autonomy (independent walking). The rate of patients living at home, the SF-8 PCS and Barthel index was higher in the operative group. The improvement of autonomy between t1 and t4 was significant in the operated group (*p* = 0.04) but not in the conservative group (*p* = 0.96).

**Table 5:** Patient-reported outcomes of conservatively (*n* = 48) and operatively (*n* = 48) treated patients with FFP types II–IV during the course of the observation period

	Before trauma	t1	t2	t3	t4
Patients living at home independently or with assistance (%)					
FFP types II–IV conservative	83.3	*n.a*.	37.5	79.4	86.2
FFP types II–IV operative	87.5	*n.a*.	44.7	86.7	92.3
Short Form-8 Physical Component Score (median)					
FFP types II–IV conservative	41.09	*n.a*.	23.71	34.95	34.8
FFP types II–IV operative	35.77	*n.a*.	23.91	31.23	39.63
Short Form-8 Mental Component Score (median)					
FFP types II–IV conservative	57.25	*n.a*.	52.52	57.48	56.68
FFP types II–IV operative	55.83	*n.a*.	55.43	55.46	55.61
European Quality of Life - 5 Dimensions - 5 Levels (median)					
FFP types II–IV conservative	0.9	0.17	0.35	0.76	0.7
FFP types II–IV operative	0.85	0.14	0.33	0.76	0.7
Patients walking independently (%)					
FFP types II–IV conservative	*n.a*.	27.1	4.2	35.3	27.6
FFP types II–IV operative	*n.a*.	12.5	6.4	15.6	30.8
Barthel index (median)					
FFP types II–IV conservative	*n.a*.	45	60	85	87.5
FFP types II–IV operative	*n.a*.	45	75	85	95
Parker Mobility Score (median)					
FFP types II–IV conservative	*n.a*.	1	2	4	5
FFP types II–IV operative	*n.a*.	1	2	4	6
Numeric Rating Scale on load (median)					
FFP types II–IV conservative	*n.a*.	10	8	3	2
FFP types II–IV operative	*n.a*.	9	5	5	5

*n.a.* not available

### FFP type II conservative and operative

59 patients suffered FFP type II. Mean age was 80.2 years (SD 9.6 years). There were 51 women (86.4%) and 8 men (13.6%). 51 patients lived independently or with assistance in their own home (86.5%). 45 were treated conservatively (76.3%), 14 operatively (23.7%). Demographics and selected parameters of patients with FFP type II during hospitalization and during the course of the observation period are depicted in Table 6. The evolution of the patient-reported outcomes is depicted in Table 7. There were some important differences between the conservative and operative group. Operatively treated patients were 3 years younger than the conservatively treated (77.9 versus 80.9 years) (*p* = 0.19), had more often osteoporosis in their medical history (78.6% versus 51.1%) (*p* = 0.07) and presented much later after the onset of complaints (15.5 days versus 0 days) (*p* = 0.002). Before trauma and at t1, SF-8 PCS and EQ-5D-5L were lower in the operative group and autonomy of the operatively treated patients was more severely restricted than of the conservatively treated (7.1% walked without aid versus 28.9%). Operative patients were operated at a median of 8 days after admission. Median LoS of the operative group was 17 days versus 9 days in the conservative group (*p* < 0.001). There were slightly more general in-hospital complications in the operative group (23.1%) than in the conservative group (17.8%) but without significant difference (*p* = 0.69). There were surgery-related complications in 2 patients (15.4%) but no surgical revisions needed. Four patients of the conservative group (8.9%) underwent secondary operative treatment because of fracture progression. One-year mortality rate was 14.5% in the conservative and 7.3% in the operative group (*p* = 0.548). At t4, 85.2% of the conservative group and 81.8% of the operative group lived at home. 29.6% of the conservative and 27.3% of the operative group could walk independently. Whereas the rate of patients with full autonomy (independent walking) did not change between t1 and t4 (28.9% at t1 and 29.6% at t4) in the conservative group, this rate changed importantly in the operative group (7.1% at t1 and 27.3% at t4).

**Table 6:** Demographics, type of treatment and selected data of conservatively and operatively treated patients with FFP type II during the course of the observation period

	All patients with FFP type II	FFP type II conservative treatment	FFP type II operative treatment	*p* value
Number (%)	59 (100)	45 (76.3)	14 (23.7)	
Mean age (years)	80.2	80.9	77.9	0.19
Standard Deviatio*n* (SD)	9.6	9.7	9.1	
Women; *n* (%)	51 (86.4)	38 (84.4)	13 (92.9)	0.67
Men; *n* (%)	8 (13.6)	7 (15.6)	1 (7.1)	0.67
Patients with comorbidities; *n* (%)	53 (89.8)	40 (88.9)	13 (92.9)	1
Patients with two comorbidities or more; *n* (%)	32 (54.2)	26 (57.8)	6 (42.9)	0.33
Osteoporosis in medical history; *n* (%)	34 (57.6)	23 (51.1)	11 (78.6)	0.07
Previous osteoporotic fracture, *n* (%)	21 (35.6)	14 (31.1)	7 (50.0)	0.22
Patients with more than 5 different drugs per day; *n* (%)	41 (69.5)	32 (71.1)	9 (64.3)	0.74
Anti-resorptive medication; *n* (%)	19 (32.2)	14 (31.1)	5 (35.7)	0.75
**Living situation before admission**; *n* (%)				
Independently	44 (74.6)	35 (77.8)	9 (64.3)	0.31
Assisted living	7 (11.9)	4 (8.9)	3 (21.4)	0.34
Nursing home	5 (8.5)	4 (8.9)	1 (7.1)	1
Hospital	3 (5.1)	2 (4.4)	1 (7.1)	0.56
**Trauma mechanism**; *n* (%)				
Fall from standing position	42 (71.2)	34 (75.6)	8 (57.1)	0.14
Recurrent falls	3 (5.1)	3 (6.7)	0 (0.0)	0.14
No trauma memorable	14 (23.7)	8 (17.8)	6 (42.9)	0.14
**Time between trauma or beginning of complaints and t1 **(days)				
Median	1	0	15.5	**0.002**
Min	0	0	0	
Max	123	123	121	
**Median length of hospital stay** (days)	10	9	17	** < 0.001**
Min (days)	3	3	8	
Max (days)	27	26	27	
IQR (days)	7–15	5–12	14–21	
**Median length of postoperative hospital stay** (days)	*n.a.*	*n.a*.	9	
Patients with general complications; *n* (%)	11/58 (19.0)	8/45 (17.8)	3/13 (23.1)	0.69
Urinary tract infection; *n*	6	3	3	0.12
Pneumonia; *n*	4	4	0	1
Bedsore; *n*	1	1	0	1
**Patients with surgery-related complications**; *n* (%)	*n.a*.	*n.a*.	2 (15.4)	
Implant malposition; n			2	
Surgical revision; *n* (%)	*n.a*.	*n.a*.	0 (0.0)	
Secondary operative treatment; *n* (%)	*n.a*.	4 (8.9)	n.a	
One-year mortality rate; %	13.7	14.5	7.3	0.548
**Living environment at t4**; *n* (%)				
Home	32 (84.2)	23 (85.2)	9 (81.8)	0.5
Nursing home	5 (13.2)	4 (14.8)	1 (9.1)	0.5
Hospital	1 (2.6)	0 (0.0)	1 (9.1)	0–5
New osteoporotic fracture between t2 and t4; *n* (%)	6 (15.8)	2 (5.1)	4 (30.8)	**0.0286**
Re-hospitalization between t2 and t4; *n* (%)	15 (39.5)	16 (43.7)	5 (41.7)	0.721

*n.a*. not applicable, *p* values below 0.05 are depicted in bold

**Table 7:** Patient-reported outcomes of conservatively (*n* = 45) and operatively (*n* = 14) treated patients with FFP type II during the course of the observation period

	Before trauma	t1	t2	t3	t4
Patients living at home independently or with assistance (%)					
FFP type II conservative	86.7	*n.a*.	40	78.8	85.2
FFP type II operative	85.7	*n.a*.	53.8	91.7	81.8
Short Form-8 Physical Component Score (median)					
FFP type II conservative	42.05	*n.a*.	24.13	35.22	35.08
FFP type II operative	38.39	*n.a*.	21.66	27.48	39.63
Short Form-8 Mental Component Score (median)					
FFP type II conservative	57.25	*n.a*.	52.2	57.6	57.08
FFP type II operative	52.68	*n.a.*	57.13	57.54	59.78
European Quality of Life - 5 Dimensions - 5 Levels (median)					
FFP type II conservative	0.9	0.23	0.35	0.77	0.72
FFP type II operative	0.76	0.21	0.38	0.91	0.74
Patients walking independently (%)					
FFP type II conservative	*n.a*.	28.9	4.4	36.4	29.6
FFP type II operative	*n.a*.	7.1	7.7	8.3	27.3
Barthel index (median)					
FFP type II conservative	*n.a*.	45	60	85	85
FFP type II operative	*n.a*.	42.5	65	85	95
arker Mobility Score (median)					
FFP type II conservative	*n.a*.	1	2	4	5
FFP type II operative	*n.a*.	1	2	5	6
Numeric Rating Scale on load (median)					
FFP type II conservative	*n.a*.	10	8	2.5	0
FFP type II operative	*n.a*.	7.5	5	5	3

*n.a.* not available

### FFP type III

Eleven female patients suffered FFP type III. Mean age was 78.1 years (SD 12.4 years). Nine patients lived independently or with assistance in their own home (81.8%). Only one patient was primarily treated conservatively (9.1%) but needed secondary surgery due to fracture progression. Ten patients were primarily treated operatively (90.9%). Demographics and selected parameters of patients with FFP type III during hospitalization and during the course of the observation period are depicted in Table 8. The evolution of the patient-reported outcomes is depicted in Table 9. The patients presented at an average of 6.5 days after the onset of complaints and were operated at a median of 5 days after admission. The LoS was 14 days. There were general in-hospital complications in 4 patients (40%), mainly due to urinary tract infection, but there was no surgery-related complication. The 1-year mortality rate was 6.7%. At t4, all remaining patients lived at home (100%). Whereas at t4, EQ-5D-5L was lower than in patients with FFP type II, SF-8 PCS, SF-8 MCS and Barthel index were comparable.

**Table 8:** Demographics and selected data of operatively treated patients with FFP type III and FFP type IV during the course of the observation period

	FFP type III operative	FFP type IV operative
Number	10	24
Mean age (years)	77.7	75.8
Standard deviatio*n* (SD)	12.9	8.6
Women; *n* (%)	10 (100.0)	24 (100.0)
Patients with comorbidities; *n* (%)	10 (100.0)	19 (79.2)
Patients with two comorbidities or more; *n* (%)	6 (60.0)	13 (54.2)
Osteoporosis in medical history; *n* (%)	4 (40.0)	16 (66.7)
Previous osteoporotic fracture, *n* (%)	3 (30.0)	10 (41.7)
Patients with more than 5 different drugs per day; *n* (%)	6 (60.0)	17 (70.8)
Anti-resorptive medication; *n* (%)	1 (10.0)	12 (50.0)
**Living situation before admission**; *n* (%)		
Independently	9 (90.0)	20 (83.3)
Assisted living	0 (0.0)	1 (4.2)
Nursing home	1 (10.0)	1 (4.2)
Hospital	0 (0.0)	2 (8.4)
**Trauma mechanism**; *n* (%)		
Fall from standing position	7 (70.0)	10 (41.7)
Recurrent falls	1 (10.0)	3 (12.5)
No trauma memorable	2 (20.0)	11 (45.8)
**Time between trauma or beginning of complaints and t1** (days)		
Median	6.5	41
Min	0	0
Max	252	361
**Median length of hospital stay** (days)	14	14
Min (days)	9	7
Max (days)	28	30
IQR (days)	10.25–17	9–20
**Median length of postoperative hospital stay** (days)	9	8.5
**Patients with general complications**; *n* (%)	4 (40.0)	9 (37.5)
Urinary tract infection; *n*	3	7
Pneumonia; *n*	0	4
Bedsore; *n*	2	0
**Patients with surgery-related complications**; *n* (%)	0 (0.0)	
Implant malposition with paresis; *n*		1
Plate breakage	2 (8.3)	1
**Revision surgery**; *n* (%)	0 (0.0)	1 (4.2)
ne-year mortality rate; %	6.7	9.1
**Living environment at t4**; *n* (%)		
Home	8 (100.0)	19 (95.0)
Nursing home		1 (5.0)
New osteoporotic fracture between t2 and t4; *n* (%)	0 (0.0)	3 (13.6)
Re-hospitalization rate between t2 and t4; *n* (%)	2 (20.0)	8 (35.2)

*n.a.* not available

**Table 9:** Patient-reported outcomes of patients with FFP type III and type IV during the course of the observation period

	Before trauma	t1	t2	t3	t4
Patients living at home independently or with assistance (%)					
FFP type III	90	*n.a*.	30	70	100
FFP type IV	87.5	*n.a*.	45.8	91.3	95
Short Form-8 Physical Component Score (median)					
FFP type III	44.93	*n.a*.	22.99	33.53	36.9
FFP type IV	27.53	*n.a*.	25.39	31.23	39.35
Short Form-8 Mental Component Score (median)					
FFP type III	58.05	*n.a*.	63.7	56.49	54.28
FFP type IV	56.13	*n.a*.	52.95	54.79	53.79
European Quality of Life - 5 Dimensions - 5 Levels (median)					
FFP type III	0.88	0.16	0.18	0.39	0.55
FFP type IV	0.87	0.1	0.38	0.73	0.74
Patients walking independently (%)					
FFP type III	*n.a*.	10.0	0.0	10.0	37.5
FFP type IV	*n.a*.	16.7	8.9	21.7	30.0
Barthel index (median)					
FFP type III	*n.a*.	37.5	55	67.5	85
FFP type IV	*n.a*.	45	77.5	90	95
Parker Mobility Score (median)					
FFP type III	*n.a*.	1	1	4	5
FFP type IV	*n.a*.	2	2	5	6.50
Numeric Rating Scale on load (median)					
FFP type III	*n.a*.	10	7.5	3.50	4
FFP type IV	*n.a*.	10	5	5	6

*n.a.* not available

### FFP type IV

**Fig. 3:**
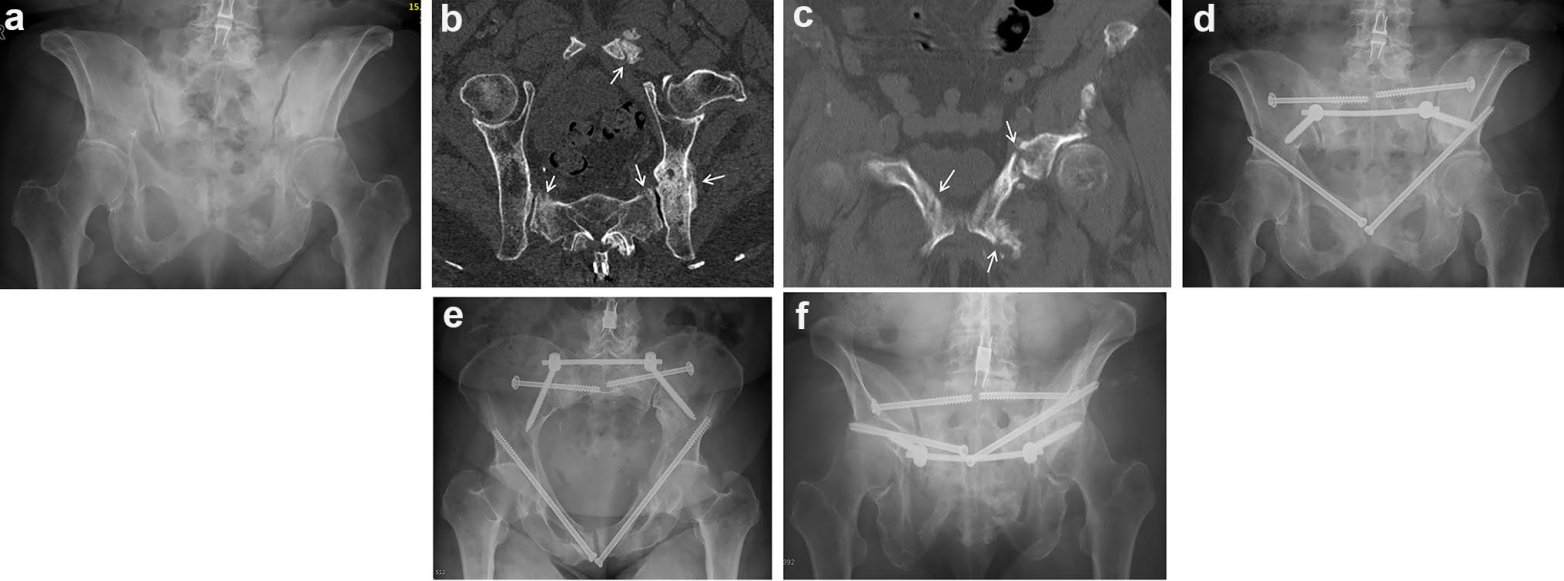
**a–f** A seventy-three-year-old female suffered of chronic pain in the pelvic region after a fall. There has been a conservative treatment for several months with pain therapy and mobilization. The a.p. pelvic overview after 6 months shows bilateral anterior pubic rami fractures with callus formation but without healing (**a**). The CT-reconstruction in the plane of the pelvic brim showed bilateral sacral ala fractures, a healed left posterior ilium fracture and a left-sided anterior instability (arrows) (**b**). Coronal CT-cut through the anterior pelvic ring showed bilateral anterior pubic rami fractures with callus formation but without healing (arrows) (**c**). These fractures corresponded with a FFP type IVc. Postoperative a.p. pelvic overview (**d**). The posterior instabilities were fixed with a transiliac internal fixator and bilateral iliosacral screws in S1. There was not enough place for safe placement of a transsacral bar in S1. The anterior instabilities were transfixed with two retrograde transpubic screws. Postoperative pelvic inlet view (**e**). Postoperative pelvic outlet view (**f**)

Twenty-six female patients suffered FFP type IV. Mean age was 76.1 years (SD 8.9 years). 22 patients lived independently or with assistance in their own home (84.6%). Two patients were treated conservatively (7.7%) and 24 patients operatively (92.3%) (Fig. 3a–f). All patients presented with comorbidities, 16 had osteoporosis in their medical history (66.7%). Demographics and selected parameters of patients with FFP type IV during hospitalization and during the course of the observation period are depicted in Table 8. The evolution of the patient-reported outcomes is depicted in Table 9. Patients with FFP type IV presented at a median of 41 days after the onset of complaints. Patients were operated at a median of 5 days after admission, and their median LoS was 14 days. There were general in-hospital complications in nine patients (34.6%), mainly because of urinary tract infection. A surgery-related complication was present twice (8.3%). One patient needed removal of an iliosacral screw because of malposition and paresis (4.2%). One-year mortality rate was 9.1%. At t4, 95% of the operated patients lived at home. Whereas only 16.7% walked independently at t1 (16.7%), 30.0% walked independently at t4. There was a steady improvement in all patient-reported outcomes between t1 and t4. The ultimate values were comparable with operatively treated patients with FFP type II and type III. Nevertheless, there was a higher sensation of pain on load.

## Discussion

In this prospective study, we searched for key factors, which influence treatment strategy and outcome in patients with FFP. We could include 110 patients in a 2-year period.

Characteristics of included patients were similar to the data of other studies: FFP-patients are of old age, the large majority being females [7–9, 14, 23] and presenting with comorbidities. More than half of our patients suffered from osteoporosis and more than one-third suffered another osteoporotic fracture before FFP although only one third (34.5%) had an antiresorptive medication at first presentation. Most patients lived independently or with assistance in their own environment. All patients with FFP type I as well as 76.3% of the patients with FFP type II were primarily treated conservatively. 91.9% of patients with FFP type III–IV were treated operatively. The mean age of operated patients was 4.1 years younger than of conservative patients (*p* = 0.04). We find comparable data in the series of Oberkircher et al. [15], Gericke et al. [24] and Höch et al. [25]. Lower age may have played a role in favor of surgical treatment in these trauma centers.

The most important finding in our study is that those patients, who ultimately received an operative treatment, had more unstable FFP types, were hospitalized later after the onset of their complaints and presented in a worse clinical form due to FFP. These conditions can be regarded as key factors in favor of operative treatment. Patients, who received surgical treatment, were admitted at a median of 1 month (33.5 days) after the onset of complaints whereas the median day of admission of the conservative group was the day of trauma (*p* < 0.001). In FFP type II, there was a difference of 15.5 days, in FFP type III of 6.5 days and in FFP type IV of 41 days. These patients received a conservative outpatient treatment by family doctors or orthopedists and were later hospitalized. At t1, the loss of autonomy of the operated group was importantly larger than that of the conservative group: only 12.5% of the operated group could walk without aid versus 27.1% of the conservative group. One year later, at t4, we see that autonomy in the conservative group did not ameliorate whereas autonomy of the operated patients was significantly better. From these data, we can conclude that patients, who present late after FFP and are hospitalized in a reduced clinical condition benefit from surgical treatment.

Especially in FFP type II patients, there is an ongoing debate on when operative treatment is beneficial. In our series, the operative FFP type II group had less autonomy (independent walking) and lower EQ-5D-5L, SF-8 PCS and Barthel index scores before and at hospital admission than the conservative group. The surgical procedure was merely performed at a median of 8 days after admission with the consequence of a longer LoS (*p* < 0.001). General in-hospital complications were slightly higher in the operative group without significant difference with the conservative group (*p* = 0.69). One year after primary presentation (t4), the EQ-5D-5L and SF-8 MCS scores were similar between the conservative and the operative group, whereas the SF-8 PCS and Barthel index were better for the operative group. One-year mortality rate of the operated patients (7.3%) was only half the value of the conservative group (14.5%), although not significant (*p* = 0.548). These data suggest that patients with FFP type II, who present later in a reduced clinical condition should be operated early after hospital admission. Data from selected outcome scores show that they recover better than their conservatively treated counterparts.

Also, in patients with FFP type III and type IV, surgical treatment proved to be safe and beneficial. All but one patient with FFP type III have been treated operatively. There were no surgery-related complications. One-year mortality rate was 6.7%, which is lower than in patients with FFP type III in the retrospective series of Rommens et al. [23]. At t4, the median values of EQ-5D-5L and SF-8 PCS nearly reached the level before FFP. The median scores of the Barthel index and the PMS, the level of autonomy and the rate of patients returning home improved steadily over time and were the highest at t4. Patients with FFP type IV had an equivalent course. It is remarkable that the operative group within the patients with FFP type IV was admitted in hospital only 6 weeks after the beginning of complaints. 45.8% of them did not remember a traumatic event. We believe that these patients primarily suffered a FFP with a lower level of instability. In the weeks before admission, insidious fracture progression led to FFP type IV [26]. The time period before hospital admission can be regarded as a period of unsuccessful conservative treatment. The median values of EQ-5D-5L and SF-8 PCS increased after operative treatment to reach their highest values at t4. Similarly, all other scores increased over time and were the highest at t4. One-year mortality was rate was 9.1%, which is comparably low as in operated patients with FFP type II and FFP type III. This data support operative treatment in these subgroups of patients with the highest degrees of pelvic instability.

This is the first study, which prospectively assesses conditions, which influence outcome after FFP. Consequently, data are not completely comparable with other published series [7, 23, 24, 27, 28]. Several studies find a longer LoS in operatively treated patients: 16.3 days for the operative versus 8.9 days for the conservative group in the series of Oberkircher et al. [15]; 8.9 days for the conservative group, 16.6 days for the patients with percutaneous and 19,3 days for the patients with open surgical procedures in the series of Gericke et al. [24]; 12.7 days for the non-operative versus 23.6 days for the operative group in the series of Schmitz et al. [29]. The rate of general in-hospital complications is important in all series with a tendency for a higher rate in the operative group. Gericke et al. mention 21.8% in the conservative group, 28.4% in the percutaneous and 33.0% in the open surgery group [24]. Schmitz et al. mention 18% for the conservative versus 34% for the operative group [29]. Osterhoff et al. calculated 34.5% for the operative versus 17.1% for the conservative group [9]. Schuetze et al. presented a rate of 21.5% in a purely operatively treated group of 116 patients [30]. One-year mortality rate in our study population was lower than in all other series, although higher than in the reference population [31].

A remarkable finding was that 57.3% of our patients were diagnosed with osteoporosis and 36.4% already suffered another osteoporotic fracture before FFP. Only 34.5% had antiresorptive medication. In the first year after hospitalization, 14.7% suffered another osteoporotic fracture. These data support our view that FFP must be regarded as an index fracture for osteoporosis and start or continuation of anti-osteoporosis therapy is needed in all these patients [32].

This study has several limitations. Although prospective, this is not randomized. Statistical comparison was only possible in some subgroups. After 1 year, there was a drop-out of more than 25% due to mortality, change of treatment, lost to follow-up or refusal of further participation in the study. Due to its specific study design, data cannot completely be compared with those of recent retrospective or prospective studies. Multicenter, prospective randomized studies are needed to collect more specific data on outcome of different treatment algorithms and find the best indications for operative treatment.

## Conclusion

Conservative treatment is reasonable and successful in patients with a lower degree of pelvic instability, who are hospitalized immediately after trauma. Key factors in favor of surgical treatment are fracture types with a higher degree of pelvic instability, patients with delayed presentation and who are in reduced clinical condition due to FFP at the time of hospital admission. Surgical treatment can be regarded as safe and reliable. Conversion from conservative to operative therapy is advisable when patients do not recover quickly. Minimal-invasive surgical stabilization is connected with an acceptable rate of in-hospital complications and a low rate of surgery-related complications. Operative treatment is connected with longer hospital stay. Outcome of surgical therapy is favorable. Specific functional and patient-related scores improve over time to be the highest 1 year after primary admission. Mortality in the operative patients is the lowest in the operative group. These data support the recommendations of Rommens and Hofmann, published in their classification paper.
